# Relation of Religious Coping and Depression Levels in Infertile Women

**Published:** 2020-04

**Authors:** Naemeh Honarvar, Mahsa Taghavi

**Affiliations:** Medical School, Islamic Azad University, Kazeroon Branch, Kazeroon, Iran

**Keywords:** *Correlation*, *Depression Level*, *Demographic Features*, *Infertility*, *Religious Coping*

## Abstract

**Objective:** Religious coping is known as one of the successful manners to cure depressed infertile women; however, research findings show that demographic factors (e.g., education level) have played an important role on the relationship between depression and religious coping scores. The goal of this study is to measure the influence of age, job status, and education level on both scores within Iranian infertile women.

**Method** **:** In this cross sectional study, 1000 women (mean age, 35.96; range, 26-45), who are recruited from different hospitals of Shiraz (Iran), are selected via multistage cluster sampling method. The reliability and validity of the translated versions of the questionnaires have been confirmed. The correlation coefficient (Spearman method), adjusted linear regression coefficient, and ordinal regression coefficient of demographic features with the depression scores/levels (minimal, mild, moderate, and severe) and religious coping scores are determined.

**Results: **A significant negative correlation is found between depression and religious compatibility scores in 1000 infertile women (ρ = -0.318, P = 0.000). In addition, the results have implied the existence of a significant correlation and linear relationship between religious coping and age and job status (P < 0.05). Furthermore, both correlation and ordinal regression of depression intensity with both job status and education level are found to be statistically meaningful (P < 0.05).

**Conclusion: **The negative correlation between religious coping and depression scores has implied the positive role of religious coping in protecting infertile women from depression, especially among employed women. Nevertheless, the correlation of religious coping with education level is not strong enough due to the nonuniform distribution of variables through their range.

From the time of human creation, fertility has been the key factor of human survival, which causes continuation of the human generation. In ancient Egypt, infertility has been a mystery in people’s mind (e.g., curse, spells) and, many assumptions have been proposed about why some women cannot be pregnant. Fertility and breeding are the base of Christian beliefs through medieval era, when infertile women have been cursed eternally and have even been occasionally physically punished.

By developing science through the Renaissance era, some academic viewpoints are proposed about the origination of infertility. Ahwazi (1) has been an Iranian medical practitioner, who has written a medical book, titled as “Alteb Almaleki (1100 AC)”, in which the structure and movements of uterus and the uterus cancer disease are described. Although with passage of time, the knowledge of people about infertility and its origins has been considerably increased and nobody punishes infertile women, this phenomenon is still a big deal for infertile women in all societies. Therefore, they confront it like a life-time disability. Hence, this type of feeling mostly leads to psychosocial problems (2).

From another perspective, infertility is still one of the most important problems in the medical society in a way that its prevalence has increased up to 50% from 1955 until present. At least, 12% of couples in different countries suffer from infertility (3). 

According to the latest statistics, the main risk factors of infertility are smoking, obesity (e.g., bulimia), anorexia, hyper physical activity (e.g., professional athletes), receiving a high dose of caffeine per day, age over 35 years, chronic diseases, stressful working environment, life style, nutrition, and exposing to chemical environments (4). Nevertheless, there still exist a lot of unknown factors yielding infertility. 

World Health Organization (WHO) has recognized infertility as the main challenge of reproductive health (5). According to the WHO statistics, more than 70 million couples all around the world (between 12% to 15%) suffer from infertility (6). Regarding the statistics of this organization, the maximum infertility rate (31%) is reported in Sheffield (England). However, the WHO statistics are sometime different with what the local organizations report. For instance, according to WHO, infertility rate is between 13% to 19%, in different parts of Iran in 2002 (7), while Ebn-Sina (Avicenna), as a valid Iranian medical organization, reports 20.2% (19.9% in cities and 22% in rural areas) infertility rate in Iran in 2005 (8). 

WHO has performed an investigation to measure the rank of infertility among the 12 worst experiences that a female may face with during her lifetime (9). They have conducted this research over European countries, such as Netherland, France and Belgium. They have reported that infertile women experience the fourth worst tensioning experience after (I) losing their mother, (II) losing their father, and (III) spouse infidelity (10). Hence, infertility is one of the most disappointing factors that ruins the spirit and self-confidence of young women (11). This problem becomes more sophisticated among traditional families in the third-world and even developing countries. Based on the statistics (12), families who live in rural areas, villages, and margin of big cities have higher eagerness to have more children, rather than those who live in crowded cities. Having considerable number of children in rural areas has been carved in their culture. This tendency is originated from a variety of psychosocial factors, such as expecting children to financially support their old parents and taking care of them (13). In such cultural context, even families with 1 or 2 children are somehow humiliated, and this situation becomes sometimes intolerable for infertile women (14). In addition to the mentioned cultural issues, infertile women have lower chance of sustaining their marriage and they are sometimes compelled to get a divorce (15). 

Most infertile women are depressed due to the feeling of having a life-time disability. Among the psychiatric disorders, depression has the highest prevalence among infertile women. Depression has a very long historical background in a way that Selsus has described depression as the result of black bile and has documented it in “De Medicine” in 100 B.C. Depression is diagnosed via DSM-V, as the most well-known standard criteria, and its intensity is measured via taking the Beck test (second version) exam, termed as BDI-II (16). To treat depression, several approaches have been suggested: prescribing drug, religious coping, doing sport, Yoga, and meditation. Among these techniques religious coping has empirically proved to have the best effect on their health.

Religious coping is defined as performing cognitive-behavioral methods (e.g., praying, going to holy places, believing in and trust to God as the absolute power) against stressful and catastrophic events. In the literature, it is emphasized that religious coping is recognized as an effective factor that can make the life of depressed people happy and meaningful. It can also help people toward their purposes and goals. Religious coping is one of the most effective ways to alleviate the spirit of young infertile women for curing their depression (17). Not only for infertility, but also for other unsolvable events like losing parents/children and cancer, religious coping has been shown to have a brilliant effect on the treatment process. 

Regarding the positive role of religious coping in the treatment of infertile women, researchers have been curious to quantitatively measure the effect of religious coping on the treatment of depression in terms of negative correlation coefficient or the error of a linear regression. These attempts are made among different groups of women with variety of cultural, religious, ethnical, race, and geographical features. In addition to the mentioned factors, age, education level, type of job, individual experience, family background, and the depth of religious belief can have a significant effect on the treatment process.

Goudarzian et al (18) have conducted a research to assess the correlation between positive religious coping and depression score in 380 patients (from both genders) with cancer in Iran. They found a meaningful negative correlation between these factors among those who have different types of cancer. Boadi and Asante (19) have investigated the statistical relationship between mental health and religious compatibility score in Ghanaian infertile women. Results on 150 participants indicate no significant relationship between their religious belief and their depression, anxiety and somatization. The lack of meaningful statistical relationship between these factors may be originated from the small number of participants or a group of participants who all have been referred from a specific place, where in their culture people neither believe in any religion nor believe in God. 

Khan et al (20) have conducted a research to find a significant relationship between religious coping and perceived stress in watchful women who care for geriatric people with dementia in Pakistan. Their results show a positive correlation between religious compatibility score and education level. Moreover, they find a negative correlation between religious coping score and economic situation, while no significant correlation is found between perceived stress and religious coping score. 

Wischmann et al (21) have investigated the effect of infertility on sexual relationship, partnership, and self-esteem in men and women separately. Their results exhibit that this physical inability highly affects the 3 mentioned factors in both genders. In another study, various levels of religious attitude on meaningful sense, loneliness, and happiness are evaluated in the elderly (22,23). Their observations show that religious coping can be considered as a preventive factor for their psychic problems. 

Bagheri-Nesami et al (24) carefully investigate the association between religious coping and quality of professional life among 285 nurses in 4 hospitals of Iran. Despite their low income and hard working conditions, religious coping has a positive effect on their self-esteem and quality of work (r = 0.387, P < 0.001).

The correlation between functional well-being and spiritual condition of 138 women (mean age: 28) with spontaneous premature ovarian failure is assessed in a study (25). They have found a significant positive correlation between these 2 factors. In addition, the meaning/peace score is highly correlated with functional well-being, while the faith score is not strongly related to the functional well-being index. Moreover, no significant correlation is found between their spiritual score or functional well-being and their age.

Due to the contradictory results on the achievements of past research on the relationship between depression and religious compatibility scores, the contribution of this paper is to statistically assess the effect of variety of demographic features, such as age, job status, and education level, on the depression score and religious compatibility score in a considerable group (n = 1000) of infertile women in medical centers of south of Iran. 

## Materials and Methods

This cross sectional study is conducted to statistically measure the correlation among demographic features, religious coping scale, and the depression score. To demonstrate the distribution and variance of the observations and results, we use descriptive statistical graph and Table.

***Population***

In this study, 1000 infertile women are selected via multistage cluster sampling method in terms of demographic features. We consider our sample size more than what is required to increase the generalizability of our results. The participants’ demographic features cover the full range of education levels (from illiterate to PhD), age (from 26 to 45 years), and job status (from unemployed to those employed full-time). Data are gathered from different governmental and private infertility clinics of Shiraz (Iran). Therefore, the enrolled participants in this study are from Shiraz and other southern cities of Iran. 

***Questionnaire***

We use 2 questionnaires, including the second version of Beck Depression Index (BDI-II) (12), and the Way of Religious Coping Scale (WORCS), with the validity of 95% (25). These questionnaires are designed to measure the depression score and religious coping scale, respectively.

The Beck Questionnaire includes 21 items, each having 4 choices, ranging from 0 to 3; therefore, the total BDI-II score for each participant can vary from 0 to 63. This test determines the degree or severity of depression. Psychologists mostly divide this range of values into 4 intervals, termed as minimal (0-13), mild (14-19), moderate (20-28), and severe (29-63). The WORCS questionnaire contains 40 questions, each includes 5 choices; therefore, the score of each individual varies from 0 to 160. The answer to each item determines how the participant deals with a specific stressful situation. This questionnaire measures the depth of participants’ religious belief/faith. Data collection takes about 3 months and all participants are interviewed and the interviewers fill up the 2 questionnaires according to their answers.

Both questionnaires are translated into Farsi and their translation accuracy are checked by 3 psychologists and psychiatrist professors. Afterward, the Farsi version of the questionnaires are translated back to English by 2 other professional psychologists and they are compared to the original version of questionnaires and the mismatched questions are detected and corrected. 

***Statistical Analysis***

To analyze the results of the collected data, chi-square, correlation coefficient, non-iterative linear regression, and ordinal regression are used. Also, the adjusted linear regression coefficients of the Beck and WORCS scores of participants versus demographic features are determined. To measure the correlation coefficient between the variables, Spearman method is used. Linear regression is used to check the amount of linearity between the religious coping scores and demographic features, while ordinal regression is used to measure the regression between the depression levels (minimal, mild, moderate and severe) and the demographic features. P value of 0.05 is considered as the threshold of statistical meaningfulness. Moreover, the 24th version of SPSS (SPSS Inc., Chicago, Illinois, USA) software is used to elicit the statistical results. 

## Results

First, the characteristics of the collected demographic features are demonstrated; then, the statistical correlation, linear and ordinal regression values between the mentioned psychosocial factors are presented.

***Demographic Features***

The demographic features of 1000 infertile women are recorded. The participants’ age ranged from 26 to 45 years (Mean ± Std = 35.96 ± 4.46), with median and mode of 36. Moreover, the age frequency distribution of the participants in the intervals of lower than 30, between 30-35, between 35-40, and between 40-45 are 13.2%, 30%, 40.8%, and 16%, respectively. Hence, the most frequency of infertility belongs to the interval of 35 to 40 years old. The frequency distribution of education level of participants in the intervals of below/equal to high school diploma, technician, Bachelor of Science (BSc) and Master of Science (MSc) and Philosophy Doctorate (PhD) is 21.6%, 21.6%, 42.8%, and 35%, respectively. The highest frequency level of education level belongs to those participants who have BSc. In addition, the frequency distribution of job status of the participants in terms of unemployed and employed is 50.4% and 49.6%, respectively. Moreover, the frequency distribution of employed and unemployed participants is fairly equal. Since the range of religious coping scores starts from 0 to 160, the cutoff point is set to 80 (half of the range). The frequency distribution of the participants whose religious coping exceeds 80 is 56.8% (n = 568) and those whose score is lower than 80 is 43.2% (n = 432). 

The frequency distribution of depression scores of the participants in terms of minimal (no depression), mild, moderate, and severe depression were 49.6%, 10.4%, 22%, and 18%, respectively. The depression score of each patient is filled up by an interviewer through an interview session. Also, 49.6% of infertile women have no significant depression (minimal), while the other half of population suffered from different degrees of depression, most of whom had moderate depression (22%). 

***Correlation between Demographic Features (Age, Education Level, Job Status) and Religious Coping***

**Figure 1:**
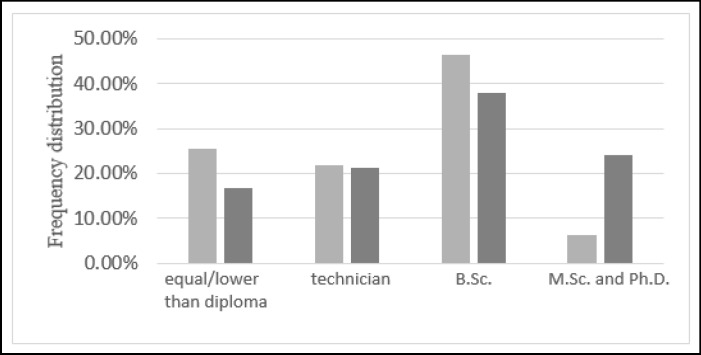
The Frequency Distribution of the Participants Versus Different Levels of Education Is Demonstrated in Figure 1. The Dark and Light Colors Show the Negative and Positive Correlation with Religious Coping Score

The correlation between age, education level, job status of the participants and their religious coping score is determined. Since the variables are continuous, their correlation coefficient (ρ) is determined with the Spearman method. The correlation coefficients of the participants’ age, education level, and job status with their corresponding religious coping score are determined (ρ = -0.137, P = 0.030), (ρ = -0.064, P = 0.316), (ρ = -0.502, P = 0.000), respectively. All the correlation coefficient values are negative, however, only those whose corresponding p value is less than 0.05 are significant. For age, the p value of 0.03 implies the reliability of this meaningful relation. Moreover, the linear regression results between religious coping and age confirms the existence of a meaningful linear relationship between these 2 variables, where the confidence interval (CI) is 95%. The correlation between education level and religious coping score shows no significant relationship between education level and religious coping score. Furthermore, the frequency distributions of the participants with positive and negative religious coping scores versus different levels of education are demonstrated in Figure 1. 

Participants with highest and lowest religious coping score have BSc degree and their frequency distribution was 46.5% and 38%, respectively. Moreover, in participants with higher education level (MSc and PhD), the religious coping score is significantly decreased, with 24.1% and 6.3% for women who have negative and positive correlation with religious coping score. To more precisely analyze the results, a linear regression method is fitted through their variations and as expected, the results do not confirm the existence of a significant linear relationship between them. 

**Figure 2:**
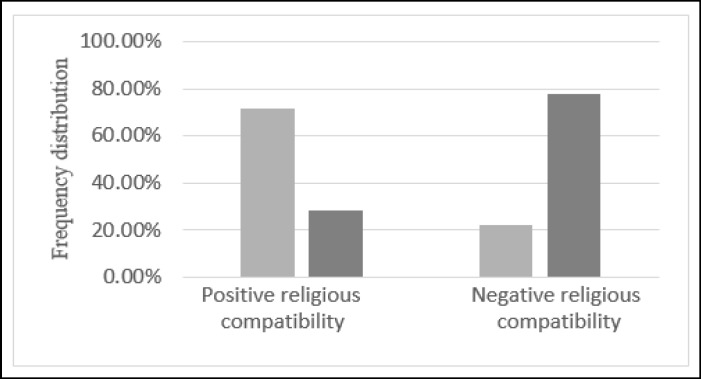
The Frequency Distribution of the Job Status of Participants versus their Religious Coping Score. The Dark Color Belongs to the Employed Group, while the Light Color Indicates the Distribution of the Unemployed Group

The relationship between participants’ job status, in terms of employed and unemployed, with the religious coping score confirms the existence of a significant correlation between these variables. In other words, the rate of participants having strong religious belief with unemployed status (housewives) is significantly (P = 0.000) higher than the employed group. The frequency distribution of job status versus religious coping is displayed in Figure 2. In addition, the regression results between these 2 factors indicate a decrease in religious coping score of the employed group.

***Relationship between the Depression Score and the Demographic Features***

The correlation between the depression score (BDI-II) and demographic features (age, education level, and job status) for the participants are expressed in Table 1. Most of psychologists consider the minimal depression level (BDI-II score: 0-13) as no depression. In other words, each healthy individual who runs BDI-II achieves a small score of depression within the rage of 0 to 13 and his/her score is not absolutely equal to zero. Therefore, the clinical signs and symptoms of depression appear for those whose BDI-II scores exceed 13. The statistical correlation coefficient and ordinal regression indicators between the individuals’ depression scores/levels and their demographic features are presented in Tables 2 and 3, respectively. The main reason for using the ordinal regression instead of linear regression for depression is that the total range of BDI-II is divided into 4 levels, including minimal (no depression), mild, moderate, and severe. To measure the regression for multilevel variables, ordinal regression method is used instead of linear regression.

The correlation between age and depression score is near zero; p* = 0.888* (Table 2) indicates that this result is not significant. In other words, by increasing the age, the depression score is not necessarily increased and no meaningful relationship is found between them. In contrast, both job status and education level parameters have a meaningful negative correlation with the depression score, where small *p *values imply the reliability of the results. This means that as the education level of infertile women increases, their depression score is decreased and similar results are achieved for the infertile women who are employed. The ordinal regression results (Table 3) of the depression levels versus the demographic features strictly confirms the correlation results (Table 2). 

***Relationship between the Depression Level and Religious Compatibility Scores***

In the last and the most important evaluation, we measure the correlation coefficients of the participants’ depression scores with their religious compatibility scores, which results in ρ = -0.318, with *P* = 0.000. The negative correlation with high reliability of the statistical test implies that infertile women with stronger religious belief have lower depression and vice versa. Table 4 illustrates the ordinal regression results between the depression score and religious coping score. In Table 4, the regression results are in the same line with the correlation results, and *P* = 0.000 indicates the high confidence and reliability of the results. 

**Table 1:** Regression Results between Religious Coping Score and Age of the Participants

**Demographic ** **Features**	**CI 95% for R**	**R**	**P value**
Age	(-2.157_-1.341)	7.751	0.000
Education Level	(-0.007_0.061)	1.546	0.123
Job Status	(-0.340_-0.221)	9.293	0.000

**Table 2:** The Correlation Coefficient and P value between the Depression Score of Participants and their Demographic Features

	**Age**	**Job Status**	**Education ** **Level**
Correlation Coefficient	ρ = 0.009, P = 0.888	ρ = -0.307, P = 0.000	ρ = -0.208, P = 0.001

**Table 3:** The Results of Ordinal Regression between the Depression Score of Participants and their Demographic Features

**Variable**	**CI 95% for OR**	**OR**	**P Value**
Age	(-0.699_1.964)	0.061	0.849
Education Level	(16.015_18.480)	115.549	0.000
Job Status	(0.418_1982)	11.685	0.000

**Table 4:** The Ordinal Regression Results for the Depression Level of Participants and their Religious Coping Score

**Variables**	**CI 95% for R**	**R**	**P Value**
Dep. Score versus Religious Comp.	(0.912_2.130)	24.618	0.000

## Discussion

In this study, the relationship between demographic features (age, education level, and job status) with the depression and the religious coping scores are assessed among 1000 infertile women. Although religious coping is known as an efficient technique to cure depression in people who have a life-time disability, its influence depends on several factors such as the depth of their religious belief, cultural background, individual experience, and the experience of their friends and relatives. It is apparent that religious coping is more common among Iranian people, who are categorized as religious, than the other nonreligious societies.

As far as everybody’s mind naturally tends to think about negative and unfinished issues, whatever these women spend their time in their loneliness, especially the unemployed, they have more free time to think about their disability; consequently, the risk of being depressed is increased among them (26). The unemployed infertile women (housewives) have stronger religious coping because most of them have grown up in religious families and they have more time to participate in religious programs, ceremonies, and even read religious books. Nevertheless, the tendency to have several children is a confounding factor that disturbs the effect of religious coping to cure these women’s depression. From another perspective, our statistics reveal that highly educated (MSc and PhD) infertile women have a lesser religious coping score, compared to those who have a lesser education level. This is because they do not have enough time to participate in religious programs and communities. This finding is in line with results of Goudarzian et al (18). The meaningful negative correlation between the depression score and religious coping score in this population emphasizes reveals an acceptable similarity between the decrease in the depression score and the increase in religious coping score. Nevertheless, confounding factors (eg, obsessional thoughts) do not allow religious coping effectively help a considerable ratio of the population against depression. For instance, the religious coping score of the highly educated group has a low correlation with a low depression score, because most of the participants in this group had a full-time job and do not have enough time to think about their disability to be depressed. The education level of the housewives’ group is lower than those women who have a full-time job. This is because knowledgeable educated people prefer to use their knowledge practically and also to financially become independent. Among the demographic features, job status is the most important factor that is correlated with the depression and religious coping scores. The regression results also confirm the significance of the meaningful linear trend between age and the religious coping score (WORCS). The correlation of age and WORCS results (ρ = -0.137) show that the relationship is not distributed uniformly over the whole range. To better interpret the results, we divide the age range into 26-30, 30-40, and >40 years and it is interesting that infertile women younger than 30 and over 40 years have a small positive correlation coefficient, while women in the range of 30 to 40 years have a larger negative correlation. Since the majority of our sample’s age ranged from 30 to 40 years, the overall correlation coefficient becomes negative. Moreover, in this study, the age range of participants is from 26 to 45 years (younger than 20 yrs.), and if our sample include more individuals in other age intervals, we would have been able to find a more correlation between age and both scores. By increase in age, people’s eagerness to religious issues is increased (27). 

Amiri et al (28) investigate the relationship between demographic features and quality of life factors among 511 infertile and 1017 fertile women. Although the quality of life scores is similarly distributed in both groups, fertile women achieve higher mean scores in mental and general health tests compared to the infertile group. Their results are in the same line with ours. Karaca and Unsal (29) find that religious and spiritual beliefs have a strong positive effect on the quality of life on infertile women in a way that these beliefs protect them against the psychosocial consequences for a group of Turkish women. Our results show a meaningful relationship between quality of life (depression level) and religious coping score; therefore, their finding is in line with our results. 

In addition, several studies have been conducted to measure the prevalence of depression among infertile women in Iran (30, 31, 32,33), most of which show a significant relationship between depression level and religious compatibility. Our statistics are consistent with the previous statistics (34,35,36). 

Meller et al (37) and Hughes et al (38) have demonstrated that infertile women, with lower social isolation, have a higher quality of life level and are also more skilled to confront with the stress and anxiety followed by infertility. This result is consistent with our achievements. In fact, the financial independency makes infertile women robust enough to protect themselves psychologically and our findings support these achievements. Domar et al (39) Andrykowski et al (40), and Underwood et al (41) emphasize that strong religious belief and spiritual health have a strong positive effect on the psychic comfort (quality of life) of infertile women. In our study, 71.4% of the participants with strong religious compatibility (score > 80) have no depression, while just 37.3% with weak religious coping have no depression. In addition, depression intensity among religious infertile women is significantly lower than the infertile women with low degree of religious coping. 

In another attempt, Aflakseir and Zarei (42) investigate religious coping strategies with stress within a considerable population of infertile women in Shiraz (Iran). They have shown an inverse significant relationship between religious coping and stress. They have also demonstrated that prolonging religious coping strategies diminishes the stress, which is in line with ours. Gharehboghlou et al (23) investigate the relationship between depression and spiritual health among nursing patients in Iran. Their achievements reveal that depression intensity is significantly decreased in patients with healthy spiritual conditions, which support our results. Taghavi et al (43) assess the effect of religious coping on women with breast cancer in Iran. Their results on 240 women show a negative correlation between depression score and religious coping score in women with strong religious beliefs who have suitable social support; their results are in line with our findings.

Hess et al (44) investigate the psychological consequences of infertility and its relationship with coping strategies in 58 infertile women in Mali, West Africa. They have examined the effect of coping techniques such as traditional and drug-based treatments, religious faith, self-isolation, and practices. They have reported that the score of 20% of them is above the psychological distress threshold and 48% of them have poor general health. They observe no significant difference between women with primary and secondary infertility. Moreover, due to cultural conditions in West Africa, participants have almost a large degree of marital tension and are humiliated by their friends and relatives. They conclude that infertile women seriously require a holistic care, covering physical, spiritual, psychological, and social needs; and their findings are consistent with our results.

## Limitation

Although the population size of 1000 is considered as an acceptable statistical population for a research project, the data are gathered from a few cities of Iran which have fairly similar culture and weather. It is evident that in those cities whose weather is mostly cloudy and foggy, the citizens are more susceptible to depression rather than the citizens of those cities with hot and sunny weather. If we have had access to the data of a much larger population, randomly gathered from hospitals of all cities of Iran, the results would have been more precise, as Iran is a vast country and its weather dramatically changes from its southern to its northern cities. From another perspective, although Iranians are mostly religious, their depth of religious belief and their corresponding religious commitment are not uniform all over this country. For instance, people who live in Yazd and Isfahan are culturally more religious than those who live in Shiraz, Tehran, or northern cities. Therefore, the results may change if we apply the same approach to demographic data of infertile women in Yazd and Isfahan cities.

## Conclusion

The results of this study support the existence of a meaningful negative correlation between religious coping and depression score. Statistical analysis results reveal that these scores are not uniformly distributed in different intervals of the demographic features. Considering other influencing factors, such as the quality of life scores in a larger population, can enhance the significance of the results.

## References

[1] Ramezanzadeh F, Aghssa MM, Abedinia N, Zayeri F, Khanafshar N, Shariat M (2004). A survey of relationship between anxiety, depression and duration of infertility. BMC Womens Health.

[2] Hafiz P, Nematollahi M, Boostani R, Namavar Jahromi B (2017). Predicting Implantation Outcome of In Vitro Fertilization and Intracytoplasmic Sperm Injection Using Data Mining Techniques. Int J Fertil Steril.

[3] Oliva A, Spira A, Multigner L (2001). Contribution of environmental factors to the risk of male infertility. Hum Reprod.

[4] Moramazi F, Roohipoor M, Najafian M (2018). Association between internal cervical os stenosis and other female infertility risk factors. Middle East Fertil Soc J.

[5] [Boivin J, Bunting L, Collins JA, Nygren KG (2007). International estimates of infertility prevalence and treatment-seeking: potential need and demand for infertility medical care. Hum Reprod.

[6] Behboodi Moghadam Z, Fereidooni B, Saffari M, Montazeri A (2018). Polycystic ovary syndrome and its impact on Iranian women's quality of life: a population-based study. BMC Womens Health.

[7] Vayena E, Rowe PJ, Griffin PD (2002). Current practices and controversies in assisted reproduction: report of a meeting on medical, ethical and social aspects of assisted reproduction, held at WHO Headquarters in Geneva, Switzerland.

[8] Vahidi S, Ardalan A, Mohammad K (2009). Prevalence of primary infertility in the Islamic Republic of Iran in 2004-2005. Asia Pac J Public Health.

[9] Balen AH (2008). Infertility in Practice.

[10] Greil AL (1997). Infertility and psychological distress: a critical review of the literature. Soc Sci Med.

[11] Masoumi SZ, Parsa P, Kalhori F, Mohagheghi H, Mohammadi Y (2019). What Psychiatric Interventions Are Used for Anxiety Disorders in Infertile Couples? A Systematic Review Study. Iran J Psychiatry.

[12] Hashemi Z, Azar IAS, Forghani F (2006). Postpartum depression and its correlates among women living in zabol (Iran).. Iran J Psychiatry.

[13] [Sarokhani D, Parvareh M, Hasanpour Dehkordi A, Sayehmiri K, Moghimbeigi A (2018). Prevalence of Depression among Iranian Elderly: Systematic Review and Meta-Analysis. Iran J Psychiatry.

[14] Ezzeddin N, Jahanihashemi H, Zavoshy R, Noroozi M (2018). The Prevalence of Postpartum Depression and Its Association with Food Insecurity among Mothers Referring to Community Health Centers. Iran J Psychiatry.

[15] Ergin RN, Polat A, Kars B, Oztekin D, Sofuoglu K, Caliskan E (2018). Social stigma and familial attitudes related to infertility. Turk J Obstet Gynecol.

[16] Kung S, Alarcon RD, Williams MD, Poppe KA, Jo Moore M, Frye MA (2013). Comparing the Beck Depression Inventory-II (BDI-II) and Patient Health Questionnaire (PHQ-9) depression measures in an integrated mood disorders practice. J Affect Disord.

[17] Vitorino LM, Marins LS, Granero Lucchetti AL, Oliveira Santos AE, Cruz JP, Oliveira Cortez PJ (2018). Spiritual/religious coping and depressive symptoms in informal caregivers of hospitalized older adults. Geriatr Nurs.

[18] Goudarzian AH, Zamani F, Nesami MB, Beik S (2017). The relationship between religious coping and depression in Iranian patients with cancer. Int J Cancer Manag.

[19] Oti-Boadi M, Oppong Asante K (2017). Psychological health and religious coping of Ghanaian women with infertility. Biopsychosoc Med..

[20] Khan ZH, Watson P, Chen Z (2012). Islamic religious coping, perceived stress, and mental well-being in Pakistanis. Archive for the Psychology of Religion.

[21] Wischmann T, Schilling K, Toth B, Rosner S, Strowitzki T, Wohlfarth K (2014). Sexuality, Self-Esteem and Partnership Quality in Infertile Women and Men. Geburtshilfe Frauenheilkd.

[22] Aliakbari Dehkordi M, Peymanfar E, Mohtashami T, Borjali A (2015). The Comparison of Different Levels of Religious Attitude on Sense of Meaning, Loneliness and Happiness in Life of Elderly Persons Under Cover of Social Welfare Organization of Urmia City. Iranian Journal of Ageing.

[23] Gharehboghlou Z, Adib-Hajbaghery M, Hajimohammad Hoseini M (2016). The Relationship between Spiritual Well-Being and Depression in Nursing Students. Iran Journal of Nursing.

[24] Bagheri-Nesami M, Kazemi A, Goudarzian AH, Nasiri F, Davari J (2017). Association between religious coping and quality of working life in nurses. Iran J Psychiatry Behav Sci.

[25] Ventura JL, Fitzgerald OR, Koziol DE, Covington SN, Vanderhoof VH, Calis KA (2007). Functional well-being is positively correlated with spiritual well-being in women who have spontaneous premature ovarian failure. Fertil Steril.

[26] Ypsilanti A, Lazuras L, Powell P, Overton P (2019). Self-disgust as a potential mechanism explaining the association between loneliness and depression. J Affect Disord..

[27] O'Brien B, Shrestha S, Stanley MA, Pargament KI, Cummings J, Kunik ME (2019). Positive and negative religious coping as predictors of distress among minority older adults. Int J Geriatr Psychiatry.

[28] Boudreaux E, Catz S, Ryan L, Amaral-Melendez M, Brantley PJ (1995). The ways of religious coping scale: Reliability, validity, and scale development. Assessment.

[29] Amiri M, Chaman R, Sadeghi Z, Khatibi MR, Ranjbar M, Khosravi A (2017). Quality of life among fertile and infertile women. Iran J Psychiatry Behav Sci.

[30] Karaca A, Unsal G (2015). Psychosocial Problems and Coping Strategies among Turkish Women with Infertility. Asian Nurs Res (Korean Soc Nurs Sci).

[31] Ramezanzadeh F, Aghssa MM, Abedinia N, Zayeri F, Khanafshar N, Shariat M (2004). A survey of relationship between anxiety, depression and duration of infertility. BMC women's health.

[32] Maroufizadeh S, Hosseini M, Rahimi Foroushani A, Omani-Samani R, Amini P (2018). The relationship between marital satisfaction and depression in infertile couples: an actor-partner interdependence model approach. BMC Psychiatry.

[33] Gdanska P, Drozdowicz-Jastrzebska E, Grzechocinska B, Radziwon-Zaleska M, Wegrzyn P, Wielgos M (2017). Anxiety and depression in women undergoing infertility treatment. Ginekol Pol.

[34] Farzadi L, Ghasemzadeh A (2008). Two main independent predictors of depression among infertile women: an Asian experience. Taiwan J Obstet Gynecol.

[35] Facchin F, Barbara G, Dridi D, Alberico D, Buggio L, Somigliana E (2017). Mental health in women with endometriosis: searching for predictors of psychological distress. Hum Reprod.

[36] Ried K, Alfred A (2013). Quality of life, coping strategies and support needs of women seeking Traditional Chinese Medicine for infertility and viable pregnancy in Australia: a mixed methods approach. BMC Womens Health.

[37] Hughes RB, Robinson-Whelen S, Taylor HB, Petersen NJ, Nosek MA (2005). Characteristics of depressed and nondepressed women with physical disabilities. Arch Phys Med Rehabil.

[38] Omani-Samani R, Maroufizadeh S, Almasi-Hashiani A, Amini P (2018). Prevalence of depression and its determinant factors among infertile patients in Iran based on the PHQ-9. Middle East Fertil Soc J.

[39] Chachamovich JR, Chachamovich E, Ezer H, Fleck MP, Knauth D, Passos EP (2010). Investigating quality of life and health-related quality of life in infertility: a systematic review. J Psychosom Obstet Gynaecol.

[40] Andrykowski MA, Bishop MM, Hahn EA, Cella DF, Beaumont JL, Brady MJ (2005). Long-term health-related quality of life, growth, and spiritual well-being after hematopoietic stem-cell transplantation. J Clin Oncol.

[41] Underwood LG, Teresi JA (2002). The daily spiritual experience scale: Development, theoretical description, reliability, exploratory factor analysis, and preliminary construct validity using health-related data. Ann Behav Med.

[42] Aflakseir A, Zarei M (2013). Association between coping strategies and infertility stress among a group of women with fertility problem in Shiraz, Iran. Journal of reproduction & infertility.

[43] Taghavi M, Kalafi E, Talei A, Dehbozorgi G, Taghavi SMA (2011). Investigating the Relation of Depression and Religious Coping and Social Support in Women with Breast Cancer. Journal of Isfahan Medical School.

[44] Hess RF, Ross R, Gililland Jr JL (2018). Infertility, Psychological Distress, and Coping Strategies among Women in Mali West Africa: A Mixed-Methods Study. Afr J Reprod Health.

